# Author Correction: Inactivation of SARS-CoV-2 and photocatalytic degradation by TiO_2_ photocatalyst coatings

**DOI:** 10.1038/s41598-022-26219-6

**Published:** 2022-12-16

**Authors:** Yun Lu, Sujun Guan, Liang Hao, Hiroyuki Yoshida, Shohei Nakada, Taisei Takisawa, Takaomi Itoi

**Affiliations:** 1 Department of Mechanical Engineering, Chiba University, Chiba, 2638522 Japan; 2 Bio-Nano Electronics Research Centre, Toyo University, Saitama, 3508585 Japan; 3 College of Mechanical Engineering, Tianjin University of Science and Technology, Tianjin, 300222 China; 4 Chiba Industrial Technology Research Institute, Chiba, 2640017 Japan

Correction to: *Scientific Reports* https://doi.org/10.1038/s41598-022-20459-2, published online 26 September 2022

The original version of this Article contained an error in the spelling of the author Taisei Takisawa which was incorrectly given as Taisei Takizawa.

The original Article and accompanying Supplementary Figures file have been corrected.

